# Examining Dementia Family Caregivers’ Forgone Care for General Practitioners and Medical Specialists during a COVID-19 Lockdown

**DOI:** 10.3390/ijerph18073688

**Published:** 2021-04-01

**Authors:** Perla Werner, Aviad Tur-Sinai, Hanan AboJabel

**Affiliations:** 1 Department of Community Mental Health, University of Haifa, Mt. Carmel, Haifa 3498838, Israel; hanan.abojabel.1987@live.com; 2 Department of Health Systems Management, The Max Stern Yezreel Valley College, Yezreel Valley 1930600, Israel; avts2309@netvision.net.il; 3 School of Nursing, University of Rochester Medical Center, Rochester, NY 14627-0446, USA

**Keywords:** aging, access, barriers, help-seeking, family caregiver, dementia, COVID-19, forgone, Andersen’s Behavioral Model

## Abstract

The present study aimed to assess dementia caregivers’ reports of the prevalence and correlates of forgone care regarding visits to a general practitioner (GP) and to a specialist during the COVID-19 lockdown in Israel, using Andersen’s Behavioral Model of Healthcare Utilization. A cross-sectional study using an online survey was conducted with 73 Israeli family caregivers of persons with dementia residing in the community (81% Jews, 86% female, mean age = 54). Overall, one out of two participants reported having to delay seeking needed help from a GP or a specialist for themselves, as well as for their relatives with dementia, during the COVID-19 lockdown period. Among the predisposing factor, education was associated with caregivers’ reports regarding forgone care for themselves as well as for their loved ones. Living with the care-receiver and income level were the enabling factors associated with forgone care for caregivers. Finally, feelings of burden were associated with caregivers’ forgone care and feelings of loneliness and perceptions of the care-receiver’s cognitive functioning were associated with care-receivers’ forgone care. Our findings show that it is essential that this population receive appropriate practical and emotional support at times of distress and crisis to enable them to continue with their caregiving role.

## 1. Introduction

Undoubtedly, the world has been shaken by the aggressive spread of the new coronavirus, COVID-19. An increasing amount of knowledge demonstrates that older people in general, and those with pre-existing conditions in particular, are at risk of developing more severe consequences when contracting the virus [1]. One group of people who have been disproportionately affected during the COVID-19 pandemic are people with dementia. Indeed, recent data show that COVID-19 mortality rates are significantly higher among people with dementia compared to those without the condition [2,3]. This finding could be attributed to the high levels of chronic comorbidities among this population [4], as well as their increased difficulties in adhering to public health recommendations, such as using a mask, social distancing, and maintaining proper hand hygiene [5]. A thorough understanding and estimation of the impact of the pandemic on people with dementia, however, cannot be attained without relating it to its effects on their caregivers.

Caregiving for people with dementia is provided primarily by unpaid family members [6] and is associated with a myriad of negative consequences for the caregiver, such as increased stress and burden, decreased quality of life, social isolation, and deterioration in the caregiver’s physical and psychological health [7]. Accordingly, several commentaries, open letters, and editorials have been published stressing the difficulties confronted by dementia family caregivers during the COVID-19 pandemic, and providing advice regarding ways to decrease them [5,8,9,10,11]. In parallel, international associations, such as Alzheimer’s Europe, are calling for the promotion of the well-being of people with dementia and their family caregivers during the crisis by providing intensive, person-centered care [12].

Defined as delaying or relinquishing a search for needed care [13], *forgone care* or unmet medical needs [14] have been noted as one of the most worrisome consequences of the COVID-19 pandemic [15,16,17]. However, no study has yet assessed the prevalence of dementia family caregivers who forgo healthcare services for themselves or for their loved ones during this crisis. This lacuna in the literature is important to fill, as studies conducted among the general adult population during COVID-19 have shown that between a third and close to half of the participants reported having to delay or relinquish seeking needed care [18,19].

Thus, the aim of the present study was to assess the prevalence and correlates of forgone care among dementia caregivers during the first COVID-19 lockdown in Israel. We examined two of the most important medical healthcare services used by people with dementia living in the community and their caregivers—visits to a general practitioner (GP) and to a specialist [20]. The research question was examined using Andersen’s Behavioral Model of Healthcare Utilization [21]. This model posits that use of healthcare services is a function of predisposing, enabling/impeding, and need factors. Predisposing factors reflect the propensity to use services and include demographic characteristics and health beliefs such as attitudes, values, and knowledge. Enabling or impeding factors reflect the resources and the ability (or lack thereof) to find and access services; health insurance is one such factor. Finally, need factors reflect perceived needs for healthcare services for specific health conditions.

## 2. Methods

### 2.1. Design and Procedure

This cross-sectional study used a purposive sample of dementia family caregivers. Potential participants were recruited through WhatsApp and Facebook social media platforms. Google Forms was used to host the anonymous and structured questionnaire, and the links to Facebook and WhatsApp were sent to associations providing support and knowledge to dementia caregivers, such as the Israeli Alzheimer’s Association. Participation was voluntary and anonymous. The data collection was initiated on 25 June 2020 and closed on 25 July 2020. The questionnaire presented a description of the aim and importance of the study, and a request that it be completed only by family members who were the main caregiver of a person with dementia. Completion of the questionnaire took approximately 10–15 min.

### 2.2. Measures

*Dependent variable—Forgone care:* Caregivers were asked whether, during the first two-month lockdown period (3 March–5 May 2020), they experienced a situation in which they had a need to visit a GP or a medical specialist for themselves or for their relative, but did not seek one out or receive an appointment. Potential responses included: didn’t have the need; had the need and used the service in person; had the need and used the service via phone or video; had the need but did not use it because the Health Maintenance Organization (HMO) providing the services cancelled it; and had the need and the service was available but decided not to use it. 

*Independent variables:* In accordance with Andersen’s model, these included predisposing, enabling/impeding, and need factors.

*Predisposing factors:* These included sociodemographic information and health beliefs.

Sociodemographic information: Caregivers were asked to report their gender, age, number of years of education, marital status (married, living with a partner, single, widowed), religion (Jewish or non-Jewish), religiosity (secular, traditional, religious, Orthodox), and employment status (employed, retired, unemployed). They were also asked to report their relative’s gender and age.

Health beliefs: These included perceived susceptibility to and fear of contracting COVID-19:

*Perceived susceptibility* was assessed with a single question: “How likely do you think it is that you will contract COVID-19?” Answers were rated on a five-point Likert-type scale, ranging from 1 = *not at all likely* to 5 = *very likely*.

*Fear* was assessed with a single question: “How much do you fear contracting COVID-19?” Answers were rated on a five-point Likert-type scale, ranging from 1 = *not at all* to 5 = *very much*.

*Enabling/impeding factors:* These included participants’ net monthly income (below average, average, and above average) and having or not having private health insurance. Additionally, similar to other studies conducted in the area of dementia [22], whether the caregiver lived with the person with dementia or not was conceptualized as part of these factors.

*Need factors:* Similar to other studies conducted in the area of dementia [22], care-receivers’ cognitive functioning and problematic behaviors and caregivers’ feelings of loneliness and burden were conceptualized as need factors. Additionally, caregivers’ reported diagnoses of chronic disease (such as diabetes and high blood pressure) were included due to being named one of the main risk factors for COVID-19.

Cognitive functioning was assessed by the Cognitive Status Scale [23]. The scale contains eight items that assess the caregiver’s perception of the care-receiver’s cognitive abilities, such as understanding simple instructions and identifying recognized individuals. Each item was rated on a five-point Likert-type scale, ranging from 0 = *not at all difficult* to 4 = *not capable at all*. An overall index was calculated by summing the items. The scale is available in Hebrew [24] and its internal reliability in this study was indicated by a Cronbach’s alpha of 0.92.

Problematic behavior was assessed using the Problematic Behavior Scale [23]. The scale contains 14 items assessing various behavioral problems, such as suspiciousness, agitation, night wandering, and anger. Caregivers were asked to report the number of days in the week preceding the interview that they had to deal with these behaviors. Each item was rated using a four-point Likert-type scale, ranging from 1 = *no problems in the previous week* to 4 = *5+ days in the previous week for all problems.* An overall index was calculated by averaging the items. The scale is available in Hebrew [24] and its internal reliability in this study was indicated by a Cronbach’s alpha of 0.87.

Feelings of loneliness were assessed with a single question: “Do you feel lonely?” Responses were rated from 1 = *never* to 4 = *all the time*. For statistical analysis, the variable was dichotomized at the median: 1 = *very often and all the time*; 0 = *not at all and sometimes*.

Feelings of burden were assessed with a single question: “Do you feel burdened because of your caregiving role?” Responses were rated from 1 = *not at all* to 5 = *very heavy burden*. For statistical analysis, the variable was dichotomized at the median: 1 = *high and very high*; 0 = *not at all or sometimes*.

### 2.3. Statistical Analyses

Descriptive statistics (means, standard deviations, percentages) were used to describe the sample and the main variables. We defined a medical service as “forgone care” if the participants reported that they did not use the needed service (in person or via phone/video), or if the provision of the service was cancelled by the HMO. Exact logistic regression was used to estimate odds ratios (ORs) of forgone care for caregivers and for care-receivers. This method was selected as being the most appropriate because of the small sample. All explanatory variables (i.e., predisposing, enabling/impeding, and need factors) were recoded as categorical variables. If not stated otherwise, they were dichotomized at the median. We assessed explanatory variables for the presence of significant multicollinearity and found none. All analyses were conducted using STATA, version 15.1 (StataCorp, College Station, TX, USA) [25].

### 2.4. Ethical Considerations

The study’s protocol was approved by the University of Haifa, Israel (reference number 253/20).

## 3. Results

### 3.1. The Israeli Context

Demographically, Israel is a young country, with only 12% of the population aged 65 and above. In Israel, the healthcare system is based on universal health coverage, with most of the basic services—such as visits to a GP or a medical specialist—provided free of charge by four Health Maintenance Organizations [26]. In terms of dementia, it is estimated that there are approximately 150,000 people in Israel with the diagnosis, with the majority being cared for in the community by their relatives.

The COVID-19 pandemic broke out in Israel in mid-February 2020. Up to 20 February 2021, 754,998 people have been infected with the virus, 5596 have died, and three general lockdowns have been conducted [27]. During lockdowns, most economic activity was halted, and the healthcare system was confronted with priority-setting dilemmas regarding the provision of health services to the population.

### 3.2. Caregivers’ and Care-Receivers’ Sociodemographic Characteristics

A total of 78 fully completed questionnaires were returned. However, in five of them the respondents stated that they were not family caregivers. Thus, analyses were performed for a sample of 73 participants. As can be seen in Table 1, the majority were female, Jewish, traditional or religious, and reported having more than 12 years of education and a below-average income. Their mean age was 54 (range 19–85) and they had an average of 14 years of education (range 7–22). Regarding the care-receivers’ characteristics, the majority were female and did not live with the caregiver (60%). Their mean age was 81 (range 61–95) and on average they were diagnosed with dementia five years previously (range 1–5). A quarter of the caregivers reported having a diagnosis of diabetes; however, only a small percentage reported having other chronic diseases. Thus, these variables were not included in further analyses.

### 3.3. Prevalence of Forgone Care for Dementia Caregivers and Care-Receivers

As can be seen in Table 2, 54% of the participants reported that they themselves had had a need to visit a GP, and 48% had had a need for a specialist. The percentages reported for their loved ones were higher: 75% reported that the care-receiver needed to visit a GP, and 65% a specialist during the lockdown period. Around half of these participants reported that they had forgone these services both for themselves and for their relative with dementia (Table 3).

### 3.4. Correlates of Forgone Care for Dementia Caregivers and Care-Receivers

Table 4 displays the results of the exact logistic regressions used to examine the determinants of forgone care for caregivers and care-receivers. Only variables that were statistically significant in bivariate analyses (data available from the authors) were included in the regressions. Several interesting results emerged from the data. First, caregivers having a higher educational level (one of the predisposing variables) were significantly less likely to report forgone care for themselves and for their loved ones compared to caregivers who had a lower level of education. Second, enabling factors were significant correlates of forgone care for the caregiver but not for the care-receiver: family caregivers living with the person with dementia, and those with a net monthly income equal to or above the average, had a significantly lower likelihood of forgone care for themselves compared with family caregivers who did not live with the care-receiver and those with net monthly incomes below the average.

Finally, among the need factors, caregivers reporting higher levels of burden had a greater likelihood of forgone care for themselves than did those reporting a lower level of burden. The likelihood of forgone care for the care-receivers was higher among those who reported higher—compared to lower—levels of loneliness, and for those who perceived their relative to have worse cognitive functioning compared to those who reported a better status for their relatives.

## 4. Discussion

Providing care to a person with dementia is stressful under ordinary circumstances; however, the COVID-19 pandemic poses new challenges and difficulties for family caregivers. Meeting all the medical needs of caregivers and care-receivers may be only one of these challenges. Although many people decide during a lockdown to forgo or delay care because of fear or because providers discontinue a service [19], the consequences of these behaviors might be more detrimental for people with dementia and their family caregivers than for others because of the stress and diminished quality of life associated with caregiving.

However, research on the topic is extremely scant. Indeed, to the best of our knowledge only two empirical studies have been published to date. The first used quantitative methods to examine to what extent 80 dementia family caregivers’ experiences of stress and behaviors were affected by the social isolation imposed by the COVID-19 protective measures [28]. The second study qualitatively explored the experiences and needs of 31 family caregivers of people with dementia [29]. Despite their methodological and cultural differences, both studies found that one of the main concerns of the caregivers was the difficulty in accessing healthcare services (such as general practitioners and specialists), a difficulty that might lead to forgone care. Our study is, to the best of our knowledge, the first to have examined the extent and correlates of forgone care for dementia family caregivers and care-receivers, using Andersen’s Behavioral Model as a theoretical framework.

Half of the participants reported having to forgo their need for receiving care from a GP or from a medical specialist for themselves as well as for their relatives with dementia. This finding is striking. First, these percentages are higher than the ones reported in a recent survey conducted among an Israeli sample of adults aged 40 and over [19] and in a survey conducted among an American sample aged 18 to 64 [30], reflecting the vulnerability of dementia family caregivers, for whom it has been demonstrated that even in normal times, higher numbers of unmet needs are consistently associated with decreased mental and physical health [31,32,33]. Second, our findings stress the importance of addressing the problem promptly, especially because the services examined—GPs and medical specialists—are the backbone of care for people with dementia living in the community and their caregivers [34].

Regarding the contribution of Andersen’s model predisposing factors to the explanation of forgone care, our study showed, similar to others [33,35], that having lower education was associated with increased forgone care for both caregivers and care-receivers. Although none of the examined enabling factors were associated with forgone care for the person with dementia, participants reporting lower levels of income had an increased likelihood to forgo care for themselves. Similar to education, this variable might be associated with an increased ability to pay for additional or alternative sources of help [36]. Previous studies in this area have shown that living arrangements are an important enabling factor, with persons with dementia living with others being associated with lower levels of unmet needs [33,37]. Findings from our multivariate analyses demonstrate that this relationship exists also for the needs of caregivers.

Finally, regarding need factors, caregivers’ feelings of burden was the only variable significantly associated with participants’ reports about forgoing GP or medical specialist services for themselves, increasing the likelihood of forgoing care by 124%. Similarly, feelings of loneliness significantly increased the likelihood of forgoing care for care-receivers by 158%, but not for caregivers. These striking findings add to the abundant literature on the negative consequences that high levels of burden and loneliness have on caregivers, and on the caregiving role [38,39], and emphasize the need to address them at times of crisis for the well-being of caregivers and care-receivers. Finally, caregivers perceiving their relatives to have lower cognitive abilities increased the likelihood of forgoing services for care-receivers from either a GP or a medical specialist by 169%. This might be a result of care-receivers having objectively more cognitive limitations, as estimated by caregivers, and needing therefore more medical attention. A similar finding was reported by Park and colleagues [40] in a study including 320 family caregivers and examining unmet needs in four care areas (environmental, physical, psychological, and social). Alternatively, it might be associated with caregivers’ increased difficulties in managing people with advanced dementia during the lockdown, as well as with their concerns about their frail and vulnerable relatives being infected by the virus if they left their homes.

### 4.1. Limitations of the Study

We have to acknowledge some limitations of our study. First, we used a rather small convenience sample. While the difficulty of recruiting caregivers of persons with dementia is well known [41], it might be even greater at times of distress and crisis, such as a lockdown, or when conducted online, which means that non-internet users (who are usually older, from lower socio-economic status, and have a more restricted network of acquaintances and support) did not have the opportunity to participate. However, it should be noted that our study sample’s distribution in terms of gender, age, and relationship to the person with dementia was identical to the characteristics reported worldwide for this population [42]. Second, a further methodological limitation is that the small size of the sample size might have contributed to low precision for some model estimates. Although we hope that the use of exact logistic regression reduced this problem, future studies using larger and more representative samples should be conducted in order to increase confidence in our preliminary results. Third, another limitation was the study’s cross-sectional design, which allows us to demonstrate associations but not to infer causal relationships. Fourth, we used single questions to assess some of the variables. Fifth, our study was based solely on caregivers’ reports. It is suggested that future studies obtain identical information from care-receivers as well, when possible [43]. Sixth, we did not collect information regarding the specific etiology of dementia. Future studies should include this information, especially since different types of dementia might affect the caregiving experience differently. Finally, we did not collect information regarding the residence of the participants.

Two additional limitations unique to the time of the study—i.e., the COVID-19 pandemic—should be noted. First, in the literature on forgone care or unmet needs, this behavior is always judged to have a negative outcome. However, at times of crisis, it might be a positive form of behavior because it decreases the risk of exposure to the virus. Lastly, we did not assess the perceived urgency of the forgone services or whether the need expressed was associated with an acute medical problem or not.

Despite its limitations, this preliminary yet pioneering study contributes important theoretical and practical knowledge to one of the most potentially deleterious consequences of the current pandemic for dementia family caregivers in different countries and cultures: delaying/forgoing healthcare services for themselves and for their relatives despite needing such services.

### 4.2. Theoretical and Practical Conclusions

Conceptually, the strength of our study lies in the use of Andersen’s Behavioral Model as its framework. We have demonstrated that this model has a good base to guide the understanding of forgone care for GPs and medical specialists in time of crisis when assessing caregivers’ needs. However, none of the examined enabling factors were significantly associated with forgone care for the person with dementia. This finding suggests that, when considering whether to renounce getting help for their relatives from these basic health services providers, family caregivers rely more on predisposing and need factors than on enabling ones. Finally, our study showed that, in addition to health conditions—i.e., care-receiver’s perceived cognitive functioning—caregivers’ distressed feelings, such as burden and loneliness, are important contributors for forgoing medical care at times of a crisis.

Practically, in light of the high prevalence of forgone medical care found among dementia family caregivers in the context of the COVID-19 pandemic, it is urgent to dealing with the phenomenon. It is essential that this population receive appropriate practical and emotional support to enable them to continue with their caregiving role, despite the difficult times we are all living through.

Given the physical, emotional and monetary costs of not receiving medical care when it is needed, it is critical that GPs and medical specialists increase their attention to dementia caregivers and care-receivers’ necessities during the current crisis. International and local dementia-related societies have published advice and tips for dementia caregiving during these challenging times [12,44,45,46]. Some of these suggestions include increasing the use of smartphones and tablets to be in contact with other people, developing a structured daily routine, embarking on activities around the house, such as listening to music, and engaging in physical activity. Increasing the use of telemedicine has been described as a potentially positive and successful way of providing various types of health services to different populations [47], including people with cognitive deterioration and their caregivers [8,48,49]. However, the introduction of these methods might be limited by technological availability and problems, as well as by ethical and legal barriers, and by decreased willingness of patients and clinicians to understand, feel comfortable with and use these methods. Thus, these suggestions have to be made available to and understood by all the GPs and medical specialists taking care of this vulnerable and high-risk population. Moreover, these professionals should be trained to assess, identify, and address caregivers with high levels of burden and loneliness, or those who will not come to the clinic because of fear of contracting the virus. Furthermore, dementia caregivers and care-receivers should be familiarized and trained in the use of alternative and innovative ways to provide healthcare (such as the use of telemedicine and other remote ways of providing care).

Finally, our preliminary study highlighted the need for further research on the topic. For example, as stated above, it is recommended to assess and compare caregivers’ perceptions about unmet needs with those of the care-receivers. Moreover, the types of medical services examined should be expanded to reflect the use (or lack thereof) of important sources of help at times of crisis, such as, social workers, psychologists, and so on. Finally, implementing the use of qualitative or mixed-method methodologies might help in attaining a more profound understanding of the sources of dementia caregivers’ forgoing of the care of GPs and medical specialists. All these directions might translate into to an increase in the well-being of dementia caregivers and care-receivers during the current pandemic.

## 5. Conclusions

Our findings demonstrated that forgone care during the COVID-19 crisis is not only a very prevalent issue among dementia family caregivers, but also a highly complex one. The variety of predisposing, enabling and need factors found to be associated with forgone care call for the need to address them jointly and immediately, without waiting for another crisis. If this results emerged in Israel, a country characterized by an effective, universal healthcare system, suggest that the problem might be even wider in other countries. Future studies, including longitudinal designs, should further examine this topic.

## Figures and Tables

**Table 1:** Descriptive statistics for independent variables (*n* = 73).

Variables	Percentage/Mean (SD)
Predisposing factors	
Caregiver gender (%)	
Male	13.70
Female	86.30
Caregiver mean (SD) age	54.27 ± 12.31
Care-receiver gender (%)	
Male	39.73
Female	60.27
Care-receiver mean (SD) age	80.73 ± 7.69
Caregiver education (%)	
12 years or less	40.85
More than 12 years	59.15
Caregiver religion (%)	
Jewish	81.43
Non-Jewish	18.57
Caregiver/care-receiver relationship (%)	
Spouse	25.3
Children	74.7
Mean (SD) susceptibility to contracting COVID-19	2.51 ± 0.93
Mean (SD) fear of contracting COVID-19	3.13 ± 1.34
Enabling/impeding factors	
Care-receiver lives with caregiver (%)	39.73
Caregiver net monthly income (%)	
Below average	62.50
Average	16.67
Above average	20.83
Private health insurance (%)	56.16
Need factors	
Caregiver reported feelings of loneliness (%)	
Very often	41.10
Sometimes/never	58.90
Caregiver reported feelings of burden (%)	
High feelings	30.14
No/light/medium feelings	69.86
Mean (SD) number of years since diagnosis	5.01 ± 2.97
Mean (SD) care-receiver’s cognitive functioning	21.45 ± 6.72
Mean (SD) care-receiver’s problematic behaviors	2.13 ± 0.69
Caregiver reported diabetes diagnosis (%)	24.64
Caregiver reported blood pressure diagnosis (%)	7.14
Caregiver reported heart disease diagnosis (%)	9.86
Caregiver reported pulmonary diagnosis (%)	5.97

**Table 2:** Need and use of healthcare services in Israel’s basket of services (%).

	Caregiver (*n* = 73)					Care-Receiver (*n* = 73)				
Type of Service	Service Not Needed	Service Needed ^a^				Service Not Needed	Service Needed ^a^			
		Service Used in Person	Service Used via Phone/Video	Delayed Using the Service	HMO Cancelled the Service		Service Used in Person	Service Used via Phone/Video	Delayed Using the Service	HMO Cancelled the Service
GP	46.38	48.65	21.62	29.73		25.37	48.00	24.00	26.00	2.00
Specialist	52.24	40.63	6.25	37.50	15.63	34.85	34.88	6.98	48.84	9.30

^a^ Percentages are calculated for those who reported needing the service for each type of medical service. HMO—Health Maintenance Organization. GP—General Practitioner.

**Table 3:** Percentage of caregivers who had to forgo care for needed medical services.

	Caregiver (*n* = 49)		Care-Receiver (*n* = 58)	
Type of Service	Did Not Forgo Care	Forgone Care	Did Not Forgo Care	Forgone Care
GP	70.27	29.73	72.00	28.00
Specialist	46.88	53.12	41.86	58.14
**At least one of these services was forgone**	**50.00**	**50.00**	**45.45**	**54.55**

**Table 4:** Exact logistic regression model for forgone care for caregivers (CG) and care-receivers (CR).

	Caregiver (*n* = 49)			Care-Receiver (*n* = 58)		
	OR	95% CI	*P*	OR	95% CI	*P*
**Predisposing factors**						
CG gender (reference male)	0.416	0.349–1.217	0.345			
CG education (reference 12 years or less)	0.678	0.214–0.888	0.024	0.319	0.067–0.529	0.014
**Enabling/impeding factors**						
CG lives with CR	0.252	0.032–0.618	0.020			
CG monthly income	0.235	0.041–0.715	0.015			
**Need factors**						
CG feelings of loneliness				1.576	1.272–2.665	0.025
CG feelings of burden	1.242	0.652–2.374	0.045			
CR perceived cognitive functioning				1.692	1.132–3.372	0.010
Model score	13.06475			8.272194		

## Data Availability

The data presented in this study are available on request from the corresponding author.
